# Investigating the Role of Assessment Method on Reports of Déjà Vu and Tip-of-the-Tongue States during Standard Recognition Tests

**DOI:** 10.1371/journal.pone.0154334

**Published:** 2016-04-21

**Authors:** Radka Jersakova, Chris J. A. Moulin, Akira R. O’Connor

**Affiliations:** 1 School of Psychology, University of Leeds, Leeds, England, United Kingdom; 2 LPNC–CNRS UMR 5105, Université Grenoble Alpes, Grenoble, France; 3 School of Psychology and Neuroscience, University of St Andrews, St Andrews, Scotland, United Kingdom; Université Lyon, FRANCE

## Abstract

Déjà vu and tip-of-the-tongue (TOT) are retrieval-related subjective experiences whose study relies on participant self-report. In four experiments (*n*s = 224, 273, 123 and 154), we explored the effect of questioning method on reported occurrence of déjà vu and TOT in experimental settings. All participants carried out a continuous recognition task, which was not expected to induce déjà vu or TOT, but were asked about their experiences of these subjective states. When presented with contemporary definitions, between 32% and 58% of participants nonetheless reported experiencing déjà vu or TOT. Changing the definition of déjà vu or asking participants to bring to mind a real-life instance of déjà vu or TOT before completing the recognition task had no impact on reporting rates. However, there was an indication that changing the method of requesting subjective reports impacted reporting of both experiences. More specifically, moving from the commonly used retrospective questioning (e.g. “*Have you experienced déjà vu*?*”*) to free report instructions (e.g. “*Indicate whenever you experience déjà vu*.*”*) reduced the total number of reported déjà vu and TOT occurrences. We suggest that research on subjective experiences should move toward free report assessments. Such a shift would potentially reduce the presence of false alarms in experimental work, thereby reducing the overestimation of subjective experiences prevalent in this area of research.

## Introduction

Tip of the tongue (TOT) and déjà vu are both subjective experiences of a temporary malfunction in the cognitive system. They are similar in that both are thought to arise from unsuccessful or partial retrieval alongside a metacognitive appraisal of this retrieval failure as jarring. More precisely, TOT is the inability to recall a piece of information combined with an awareness that this information ought to be accessible [1] whereas déjà vu is familiarity with an episode, coupled with the awareness that this familiarity is misplaced and the episode is novel [2]. Whereas these two experiences are similar in some regards, they concern different retrieval mechanisms and are characterized by fundamentally distinctive features.

A shared challenge for déjà vu and TOT research is the lack of a diagnostic, objective measure for the experience. Researchers are thus solely reliant on self-reports in order to establish whether and how often these states have been experienced, and to test critical theories about the underlying mechanisms. These reports can either be elicited after the experiment in the form of a post-experimental questionnaire [3] or during the study on a trial-by-trial basis (e.g. [4]). While these methods differ in frequency of questioning and the temporal range they cover, both are forms of retrospective questioning and take, in essence, the same form of a *yes*/*no* eliciting question; “*During the experiment/on the last trial, did you experience déjà vu/TOT?”*.

One worry is that self-reports are unreliable, and therefore contribute to results which are limited in their capacity to inform theories of subjective experiences and their prevalence. Experimental participants are not merely passive respondents; they are often motivated to confirm what they perceive as the experimental hypothesis [5] and try to be relevant by focusing their reports on what they believe the experimenter is interested in [6,7]. This is likely true for any experiment but it has also been suggested it might be a fundamental problem for research on subjective mnemonic experiences as a result of limitations in current methods [2].

For example, it was observed that the rate of reported TOT experiences for answers to general knowledge questions increased when participants were told the questions were easy as compared to when they were told they were difficult [8]. The authors of the study interpreted this finding as a social desirability effect with participants wanting to appear knowledgeable. This suggests that expectations in regard to the task can lead to changes in participants’ reporting, independent of what participants are experiencing. Related to this is the observation that déjà vu studies often observe high déjà vu reporting on control trials not meant to induce the experience. One study, across two experiments, observed déjà vu reports in 17% and 26% of critical trials as compared to 13% and 23% of control trials, indicative of a procedure, which can reliably generate déjà vu [9]. However, the difference between déjà vu reports on critical and control trials, while significant, is surprisingly small. One must ask what else about the experimental paradigm might have led to reports of déjà vu on the control trials and how this might have influenced déjà vu reports on the critical trials. If participants interpreted déjà vu merely as a sense of familiarity then a familiarity manipulation would increase déjà vu reporting on critical trials without necessarily probing déjà vu. This is supported by a recent study, which asked participants to elaborate on the nature of their self-reported, experimentally-generated déjà vu experiences; some participants expressed uncertainty as to whether what they reported during the experiment was genuine déjà vu [10]. Corroborating self-reports is not common practice but this finding suggests it should be.

It is evident that déjà vu and TOT, by their nature, necessitate a reliance on self-report both when their incidence is assessed in real-life and in the laboratory. While this is not in itself a problem, the research discussed above suggests that self-reports are sensitive to subtle sources of contamination. The aim of this study is to explore whether there are methods of framing questions aimed at eliciting self-reports about subjective memory experiences that might be more reliable than the prevailing approach. The main focus is on the reduction of so-called false alarms: affirmative responding in the absence of the studied phenomenon.

We have identified two mechanisms by which false alarms might occur. First, participants might interpret being asked about whether they are experiencing déjà vu or TOT as indicative of a normative experience that they *should* be experiencing. This would be especially the case with a participant who is unsure of what exactly she is experiencing: she might conclude that her current state is that which the experimenter is interested in, and respond affirmatively when asked. This is analogous to an acquiescence effect or positive responding regardless of question content [11]. Alternatively, it could be that the communication between the experimenter and participants about the exact nature of the studied experience is not clear. In the déjà vu study example discussed earlier with elevated response rates on control trials, the researchers also asked an independent group to define déjà vu and the majority defined it merely as familiarity [9]. Only 6 of the 92 participants gave the operational definition used by the researchers (a sense of familiarity combined with the awareness that it is false). The same question being interpreted differently by the experimenter and the participants is obviously problematic [12].

In the four experiments presented here, we investigated participants’ reports of déjà vu and TOT on a task that we have no reason to hypothesise induces either experience: continuous recognition. The absence of a critical experimental manipulation for the generation of either experience (i.e. conditions under which we were hypothesising déjà vu or TOT would be generated) allowed us to establish conditions uncontaminated by residual effects of successful experience generation–conditions which empirical déjà vu and TOT researchers are attempting to attain during baseline/control experience assessment. In Experiment 1 we manipulated the number of times participants were asked about déjà vu or TOT to establish whether report rates show a relationship with question frequency. In Experiments 2A and 2B we assessed whether the high report rates from Experiment 1 decreased in participants who were asked to bring to mind a previous ‘real-life’ experience of déjà vu or TOT prior to completing the task. In Experiment 3 we explored whether déjà vu and TOT reporting changes with the method of questioning used–instead of asking participants at regular intervals *whether* they had the given experience (retrospective report), participants were required to indicate with a button press *whenever* it occurred (free report). Finally, in Experiment 4 we investigated whether participants’ reports changed with manipulation of the definition and the name given to the experience. Together, these manipulations allowed us to explore whether déjà vu and TOT false alarms could arise (i) due to demand characteristics and acquiescence or (ii) lack of clarity of terms and agreement on their meaning.

## Experiment 1

We investigated, between-subjects, whether repeatedly questioning participants about a subjective experience of déjà vu or TOT increased reporting of these experiences. After completing a continuous recognition task, all participants were asked whether they experienced déjà vu, experienced TOT or saw any words presented in yellow (no words were presented in yellow) during the experiment. These post-experimental reports were the main variable of interest. There were four experimental conditions instantiated prior to this retrospective assessment of experimental experience. In the first condition, participants were asked about their experience of déjà vu periodically *during* the continuous recognition task (Déjà vu condition). In the second and third conditions, participants received similar questioning about the occurrence of TOT (TOT condition) and words presented in the colour yellow (Colour condition). In the fourth condition, the recognition task was uninterrupted (Control condition). We thus explored how the frequency of various reports changed in groups of participants who were asked (i) only once on completing the task (ii) repeatedly during the task and on completing the task.

### Method

#### Materials

For each participant, a different set of words was randomly selected from a pool of 2000 singular, common English nouns from the English Lexicon Project (minimum log Hyperspace Analogue to Language frequency 8.02) [13]. Each word was presented in a colour selected at random from the options black, green, red, blue or purple (no words were presented in yellow). The study was coded in JavaScript for participants to complete via an Internet browser on their Internet-enabled device.

#### Participants

Data were collected for 224 participants (56 in each condition). The participants were 94 men and 128 women (2 did not disclose gender) with mean age of 27.8 (*SD* = 9.4; 6 did not disclose age). This was an Internet based study, advertised as a memory experiment, and participants were recruited via links to the experiment on (i) the last author’s lab website, (ii) websites advertising online psychology experiments (e.g. Psychological Research on the Net) and (iii) social networking sites (e.g. Twitter and Facebook). The collected data was anonymised and stored under numerical ID not associated with identifying information. Participants were not given any compensation but memory performance feedback was provided in the form of a breakdown of their memory performance at the end of the study. All subsequent experiments used the same procedure for recruiting participants. Ethical approval for all experiments reported in this paper was granted by the University Teaching and Research Ethics Committee, University of St Andrews (Approval PS8815).

#### Procedure

Participants first completed a consent form and were asked to provide demographic information. They were then randomly assigned to one of four conditions. All participants completed a continuous recognition task in which 120 items were serially presented. These items consisted of 80 words, 40 of which were presented once and 40 of which were presented twice (repeats). Of the 40 repeats, 10 were presented with a lag of four items, 10 with a lag of eight items, 10 with a lag of 16 items and 10 with a lag of 32 items. For each item, participants made an old/new decision using the mouse.

In the Control condition, participants always proceeded directly to the next trial. In the other three conditions, every 12 trials participants were asked whether they experienced déjà vu, TOT or whether they saw any words presented in yellow (9 times throughout the experiment). After the first question they were instructed to answer only for the period since they were last asked.

In the Déjà vu condition, participants were given a standard definition of déjà vu: “*Déjà vu is a feeling of familiarity with a situation (e*.*g*. *seeing a word) combined with an awareness that this familiarity is inappropriate (i*.*e*. *you know you have not experienced the situation before”*. In the TOT condition they were similarly provided with a definition of TOT: “*The tip-of-the-tongue sensation is the failure to bring a word to mind*, *combined with partial recall of some of its characteristics (e*.*g*. *the starting letter) and the feeling that you will bring it to mind soon”*.

At the end of the recognition task, participants in all conditions completed the same post-experimental questionnaire (PEQ). They were given a definition of déjà vu and TOT (as above) and were asked to indicate (*yes* or *no*) to three questions: whether they experienced déjà vu, TOT and seeing a word presented in yellow at any point during the experiment.

*Example participant experience*: A participant assigned to the Déjà vu condition would, at the beginning of the experiment, be given a definition of déjà vu and told she would be asked periodically to report her experience of it during the experiment. Every time she completed 12 trials of the continuous recognition task, she would be asked whether she experienced déjà vu since last asked. On completion of the experiment, she would then be asked whether, at any point during the experiment, she experienced déjà vu, whether she experienced TOT, and whether she saw any words presented in yellow. All questions received a yes/no answer.

### Results and Discussion

#### Memory performance

To establish whether recognition task performance was equivalent across conditions, sensitivity (*d'*) and bias (*c*) measures were computed (see Table 1). In calculating Hit and False Alarm rates we used a correction proposed by Snodgrass and Corwin to deal with errorless responding [14]. A one-way ANOVA revealed that groups neither differed in their sensitivity, *F* < 1, nor in their bias, *F*(3,223) = 1.99, *p* = .12, *η*_*p*_^*2*^ = .026.

**Table 1:** Recognition task Sensitivity and Bias for Experiments 1 to 4.

Experiment	Condition	Sensitivity (*d'*)	Bias (*c*)
Experiment 1	Déjà vu	2.84 (.82)	.30 (.48)
	TOT	2.66 (.91)	.14 (.47)
	Colour	2.83 (.68)	.19 (.47)
	Control	2.76 (.83)	.32 (.41)
Experiment 2A	Déjà vu	2.92 (.60)	.18 (.36)
	TOT	2.74 (.82)	.24 (.40)
Experiment 2B	Déjà vu	2.57 (.92)	.37 (.46)
	TOT	2.82 (.87)	.16 (.52)
Experiment 3	Déjà vu	2.87 (.87)	.23 (.34)
	TOT	2.89 (.67)	.17 (.30)
Experiment 4	Paramnesia	3.02 (.67)	.14 (.32)
	Unpleasant Familiarity	2.99 (.54)	.23 (.38)

Means are shown, with standard deviations in parentheses.

#### Post-experimental reports of déjà vu and TOT

**Fig 1:**
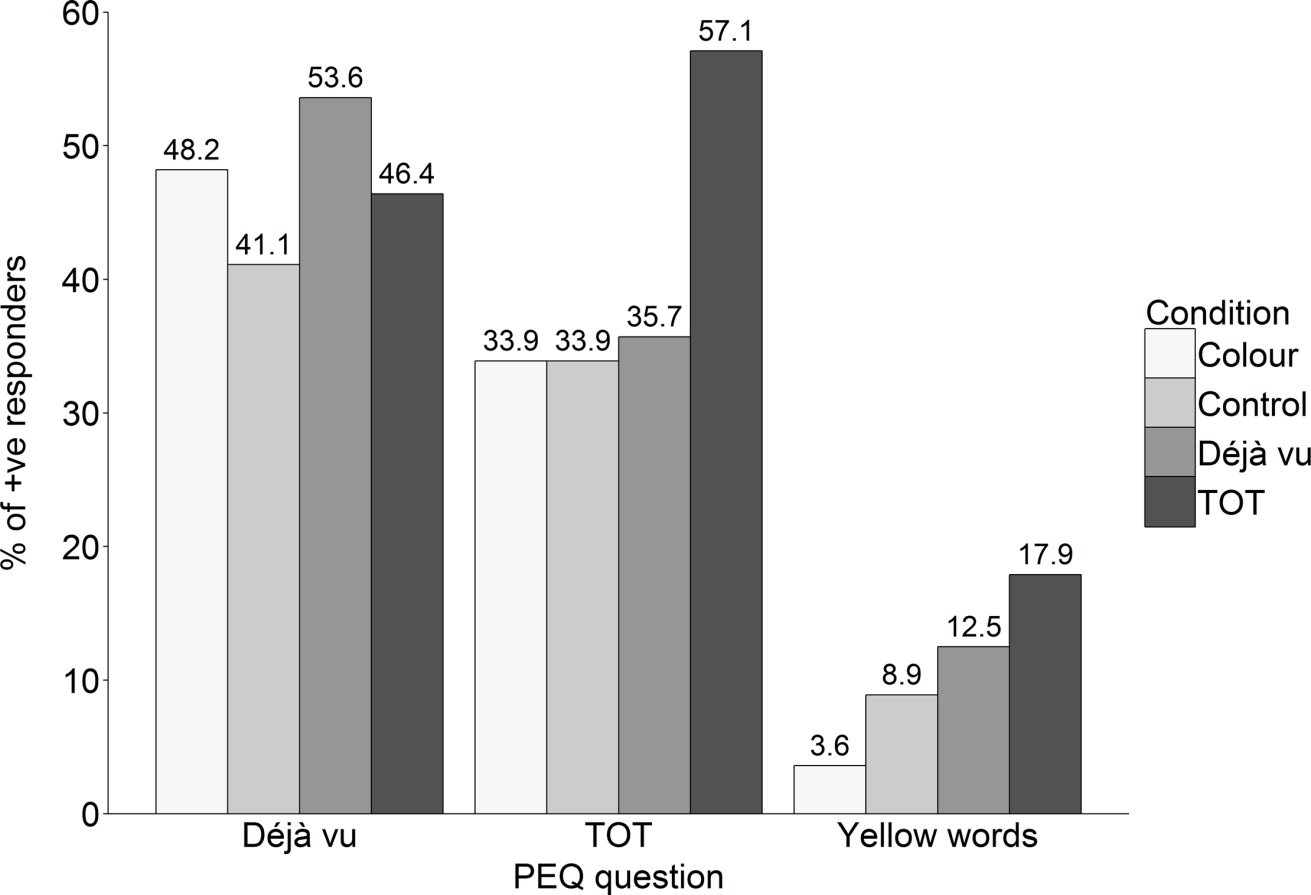
Percentage of participants reporting déjà vu, TOT and seeing yellow words in the PEQ in Experiment 1.

The principal question addressed was whether the groups differed in the percentage of déjà vu and TOT reporters. In other words, we asked whether participants were more likely to report experiencing déjà vu or TOT when asked about the experience repeatedly throughout the recognition task (Déjà vu or TOT condition) as compared to only once following the recognition task (the other 3 conditions, see Fig 1). Overall, a third to a half of participants in each group reported experiences of déjà vu and TOT. There were no differences between groups in the percentage of participants reporting déjà vu, *χ*^*2*^(3) = 1.79, *p* = .62. However, groups did differ in their reports of TOT, *χ*^*2*^(3) = 8.99, *p* < .05. Pair-wise comparisons, using the Bonferroni correction for multiple comparisons, confirmed that more participants reported experiencing TOT in the TOT group as compared to the Control and the Colour group (*p*s < .05) but not the Déjà vu group (*p* = .069).

#### Within-experiment reports of déjà vu and TOT

To further examine whether reports changed with the frequency of questioning, we analysed whether the likelihood of a within-experiment déjà vu or TOT report increased with the number of times participants were asked. Of the 9 times that participants could report an experience during the study we compared the frequency of reports for the first 3 times they were asked (i.e. the first section of the recognition task) against the intermediate and the last 3 questions. The following analyses concern only those participants who reported the given experiences in the PEQ i.e. déjà vu reporters in the Déjà vu condition and TOT reporters in the TOT condition (see Table 2). While we did not observe any difference in déjà vu reports between the three sections, *F*(2,58) = 2.78, *p* = .071, *η*_*p*_^*2*^ = .087, there was a difference in TOT reports, *F*(2,62) = 3.87, *p* < .05, *η*_*p*_^*2*^ = .11. Post-hoc tests using the Bonferroni correction showed that, TOT reports were less frequent in the first as compared to the third section (*p* < .01), neither of which differed from frequency of reports in the second section (*p*s > .29).

**Table 2:** Within-experiment reports of déjà vu (in Déjà vu conditions) and TOT (in TOT conditions) by Experiment.

Condition	Experiment	1^st^ section	2^nd^ section	3^rd^ section	Total
Déjà vu	Exp.1	1.53 (0.94)	1.87 (1.07)	1.73 (1.17)	5.13 (2.90)
	Exp. 2A	1.27 (1.04)	1.61 (1.20)	1.58 (1.25)	4.46 (3.09)
	Exp. 2B	1.53 (0.97)	2.10 (1.12)	1.73 (1.17)	5.36 (2.57)
	Exp. 3	0.38 (0.59)	0.48 (0.81)	0.86 (1.28)	1.71 (2.10)
TOT	Exp. 1	1.06 (0.91)	1.44 (1.19)	1.66 (1.18)	4.16 (2.54)
	Exp. 2A	1.00 (0.89)	1.42 (1.16)	1.58 (1.02)	4.00 (2.41)
	Exp. 2B	0.84 (0.99)	1.38 (1.09)	1.32 (1.08)	3.54 (2.52)
	Exp. 3	0.30 (0.54)	0.56 (0.84)	0.30 (0.61)	1.15 (1.23)
Paramnesia	Exp. 4	0.74 (0.94)	0.77 (0.99)	0.61 (1.15)	2.13 (2.50)
Unpleasant Familiarity	Exp. 4	0.40 (0.99)	0.80 (1.58)	0.35 (0.68)	1.55 (2.76)

Mean numbers of within-experiment reports (by section of the recognition task and total) are given for participants who reported the given experience in the PEQ. Standard deviations appear in parentheses.

#### Summary

Consistent with our predictions, we observed an increase of TOT reporting in the TOT group as compared to the Control and Colour groups and we also observed an increase in frequency of TOT reports across the recognition task with more reports toward the end than the beginning of the experiment. This pattern of results was not observed for déjà vu. An unexpected finding is that even in the Control condition, a large percentage of participants reported experiencing déjà vu (41.1%) and TOT (33.9%) suggesting a general tendency to report these experiences in the continuous recognition task. The colour reports provided a useful estimate for *yes* responding that could result from lack of attention or uncertainty about the occurrence of a phenomenon not attended to during the recognition task; they were far below the reporting observed for déjà vu and TOT. One possibility is that providing déjà vu and TOT definitions to participants does not provide adequate clarity to ensure sufficient understanding of the experience in question. Thus, in a second experiment we checked for understanding of the terms by drawing a comparison with ‘real world’ versions of the experiences.

## Experiments 2A and 2B

Given the high frequency of déjà vu and TOT reports in Experiment 1, Experiments 2A and 2B explored whether this could be due to a lack of understanding of the terms investigated, or a shift in their meaning from the real world to the laboratory. We assessed whether the frequency of déjà vu and TOT reports would change from what was observed in Experiment 1 if participants were first asked to recall a real-life, personal experience of the sensation. The aim was to make the queried experience more salient to participants while they were completing the task.

Experiment 2A and 2B used Déjà vu and TOT conditions analogous to those used in Experiment 1, with two key changes. In Experiment 2A, participants simply brought to mind and described a prior, real-life experience of déjà vu or TOT before completing the recognition task. In Experiment 2B, participants used this memory of a past experience as a reference when assessing the experimentally-generated occurrence of the experience in question. Experiment 2B thus constituted a stronger version of the manipulation in Experiment 2A. In Experiment 2B, participants also provided subjective ratings of emotionality, intensity and salience for both their real-life and experimentally-generated experiences of déjà vu and TOT. We thus investigated whether participants perceive what they report experiencing in the experiment as different to the real-life experience.

### Method

#### Participants

There were 146 participants in Experiment 2A (73 in each condition). They were 97 women, 47 men (2 did not disclose gender), mean age = 26.9 (*SD* = 9.3; 3 did not disclose age). As described in the Results, in Experiment 2A over 50% of participants did not write down a real-life experience of déjà vu or TOT when prompted to at the instructions stage. We purposefully did not force participants to respond to questions so as to avoid false reporting. However, this particular lack of compliance with instructions made it difficult to evaluate the effectiveness of our manipulation; without participants describing any experience we could not be sure they brought it to mind. As a result, in Experiment 2B we narrowed down criteria for data collection. While participants could still complete the entire study without answering any questions, data was collected only for participants who completed the subjective ratings of real-life déjà vu or TOT experiences during the instructions stage (described below). Data was collected for 127 participants (60 in the Déjà vu condition and 67 in the TOT condition), 88 female, 39 male, mean age = 30.1 (*SD* = 13.2, 2 did not disclose age).

#### Materials and procedure

The materials used were the same as in Experiment 1. As before, participants completed a continuous recognition task and after every 12 trials they were asked to indicate whether, during those 12 trials, they experienced déjà vu or TOT (Déjà vu and TOT conditions respectively). After completing the recognition task, all participants indicated in the PEQ whether they experienced déjà vu, TOT or saw any words presented in yellow (none were) at any point during the experiment.

In Experiment 2A, prior to the continuous recognition task, participants were asked to bring to mind a real-life experience of déjà vu or TOT (depending on condition) and provide a written account of this experience. In Experiment 2B, participants similarly described a past experience of déjà vu or TOT and additionally, rated its salience, intensity and emotionality with each rating made on a scale of 1 (*not at all*) to 5 (*very*). Participants in Experiment 2B were instructed to use the described, real-life experience as a point of reference for assessment of any experimentally-generated experiences. All participants who reported déjà vu or TOT in the PEQ further rated this experimentally-generated experience on salience, intensity and emotionality.

### Results and Discussion

#### Real-life déjà vu and TOT descriptions

In Experiment 2A, 58.9% participants in both the TOT condition and the Déjà vu condition did not provide any real world description, stated they could not think of any one particular instance, or indicated that it happens fairly often without elaborating. In Experiment 2B, these unelaborated responses reduced to 15.3% in the Déjà vu and 11.9% in the TOT conditions due to the substantial decrease in participants who left the question unanswered. Two participants in Experiment 2A and one participant in 2B described what they termed déjà vu-like experiences rather than an actual occurrence of déjà vu. In Experiment 2A there were two participants and in Experiment 2B one participant who reported never having experienced déjà vu. We did not exclude these participants from the following analyses, as we did not have a way of checking whether this could also be true of participants in Experiment 1, against whom we were comparing the data.

#### Memory performance

As in Experiment 1, we compared memory performance between the Déjà vu and TOT groups (see Table 1). In Experiment 2A, there was no difference between the TOT and Déjà vu conditions in sensitivity, *t*(144) = 1.54, *p* = .13, *d* = .25, or bias, *t* < 1. In Experiment 2B, the two conditions likewise did not differ in sensitivity, *t*(125) = 1.57, *p* = .12, *d* = .29, but there was a difference in bias, *t*(125) = 2.36, *p* < .05, *d* = .43, with participants more liberal (more likely to label an item as *old*) in the TOT as compared to the Déjà vu condition.

#### Post-experimental reports of déjà vu and TOT

**Fig 2:**
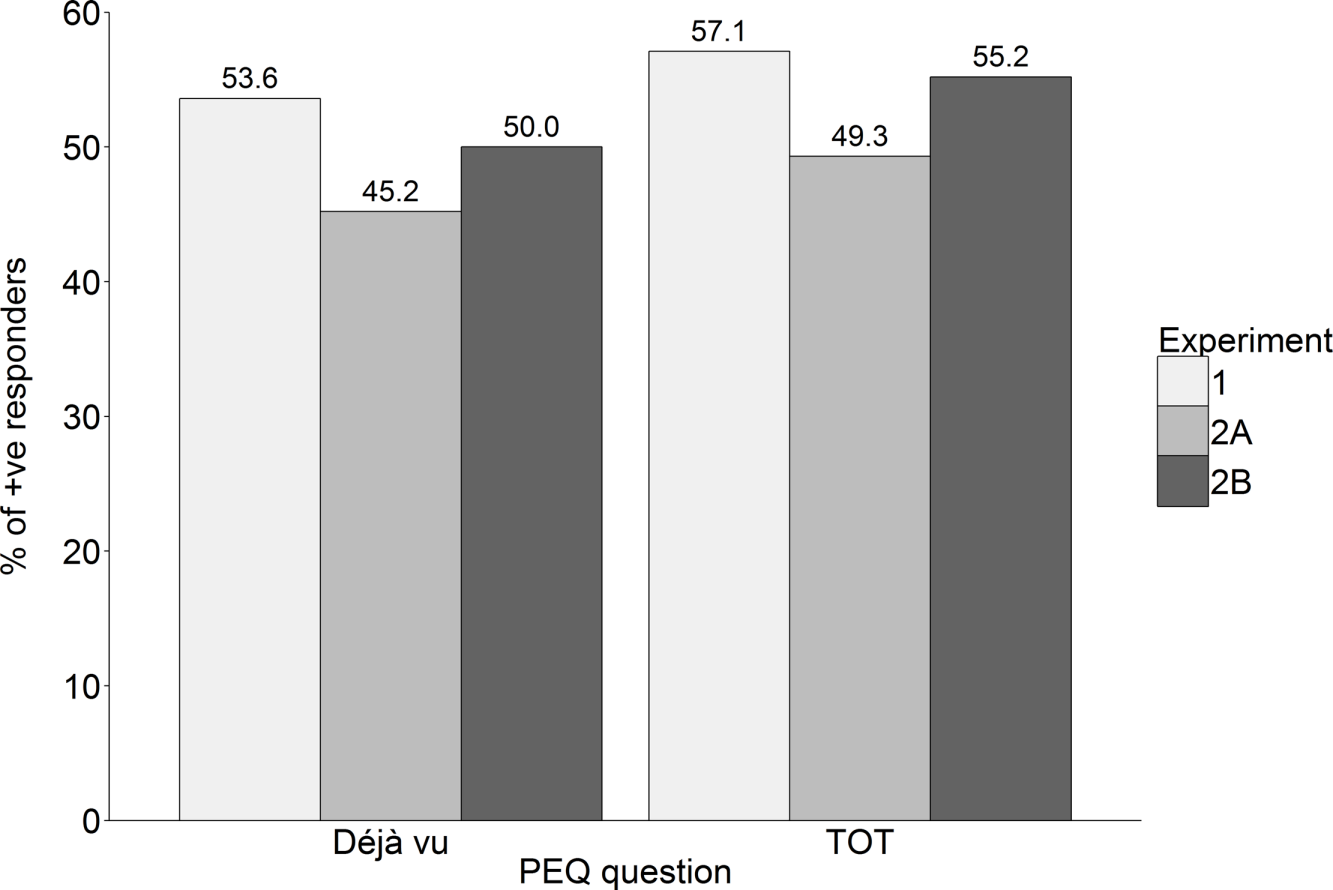
Percentage of participants reporting déjà vu and TOT in the PEQ in Experiments 1, 2A and 2B. Déjà vu reports are from Déjà vu conditions and TOT reports are from TOT conditions.

Our primary aim was to see whether the comparison with real-life experiences would influence the percentage of participants reporting subjective experiences. We compared déjà vu reports in the Déjà vu conditions of Experiment 2A and 2B with déjà vu reports in the Déjà vu condition of Experiment 1. We likewise compared TOT reports between the TOT groups of Experiment 1, 2A and 2B (see Fig 2). *χ*^*2*^ tests revealed that the percentage of déjà vu and TOT reporters across the three groups did not differ (*p*s > .63).

#### Within-experiment reports of déjà vu and TOT

While the percentage of participants reporting déjà vu and TOT in Experiments 2A and 2B was not different from Experiment 1, it is possible that the total number of experiences reported by these participants was reduced. Amongst participants who post-experimentally reported experiencing déjà vu or TOT, we analysed how many times they reported the given experience during the experiment (see Table 2). A between-subjects ANOVA revealed there was no difference in the total number of déjà vu reports for participants in the Déjà vu conditions of Experiment 1, 2A and 2B, *F* < 1. The same analysis on the number of TOT reports across the TOT groups in Experiments 1, 2A and 2B likewise revealed no significant difference, *F* < 1.

To confirm results of Experiment 1, we analysed whether, of the 9 times they were asked, participants were less likely to report an experience in the first section (on questions 1–3) as compared to the second (questions 4–6) or third (questions 7–9) section of the recognition task. While reporting did not differ across the three sections in the Déjà vu condition of Experiment 2A, *F*(2,64) = 2.53, *p* = .09, *η*_*p*_^*2*^ = .073, there was a difference in the TOT condition of Experiment 2A, *F*(2,70) = 5.23, *p* < .01, *η*_*p*_^*2*^ = .13. Post-hoc analysis using the Bonferroni correction confirmed this difference was due to reports being more frequent in the third as compared to the first section (*p* < .01) whereas there was no difference between the first and second section (*p* = .09). A similar result was observed for the TOT condition of Experiment 2B, *F*(2,72) = 5.44, *p* < .01, *η*_*p*_^*2*^ = .13, with fewer reports in the first section as compared to the second and third (*p*s < .05) sections. In Experiment 2B there was also a difference in déjà vu reports, *F*(2,58) = 3.56, *p* < .05, *η*_*p*_^*2*^ = .11. However, this consisted of more reports in the second as compared to the first section (*p* < .05) with no difference between the first and third sections (*p* = 1.0). Across all groups, there were no differences in reporting between the second and the third section (*p*s > .23). Overall, the increase in number of TOT reports with the increase in number of times participants were queried about the experience mimics the results of Experiment 1 and lends support to the original finding that the frequency of questioning can impact reports of subjective memory experiences.

#### Phenomenological characteristics of real-life vs. experimentally-generated experiences

**Fig 3:**
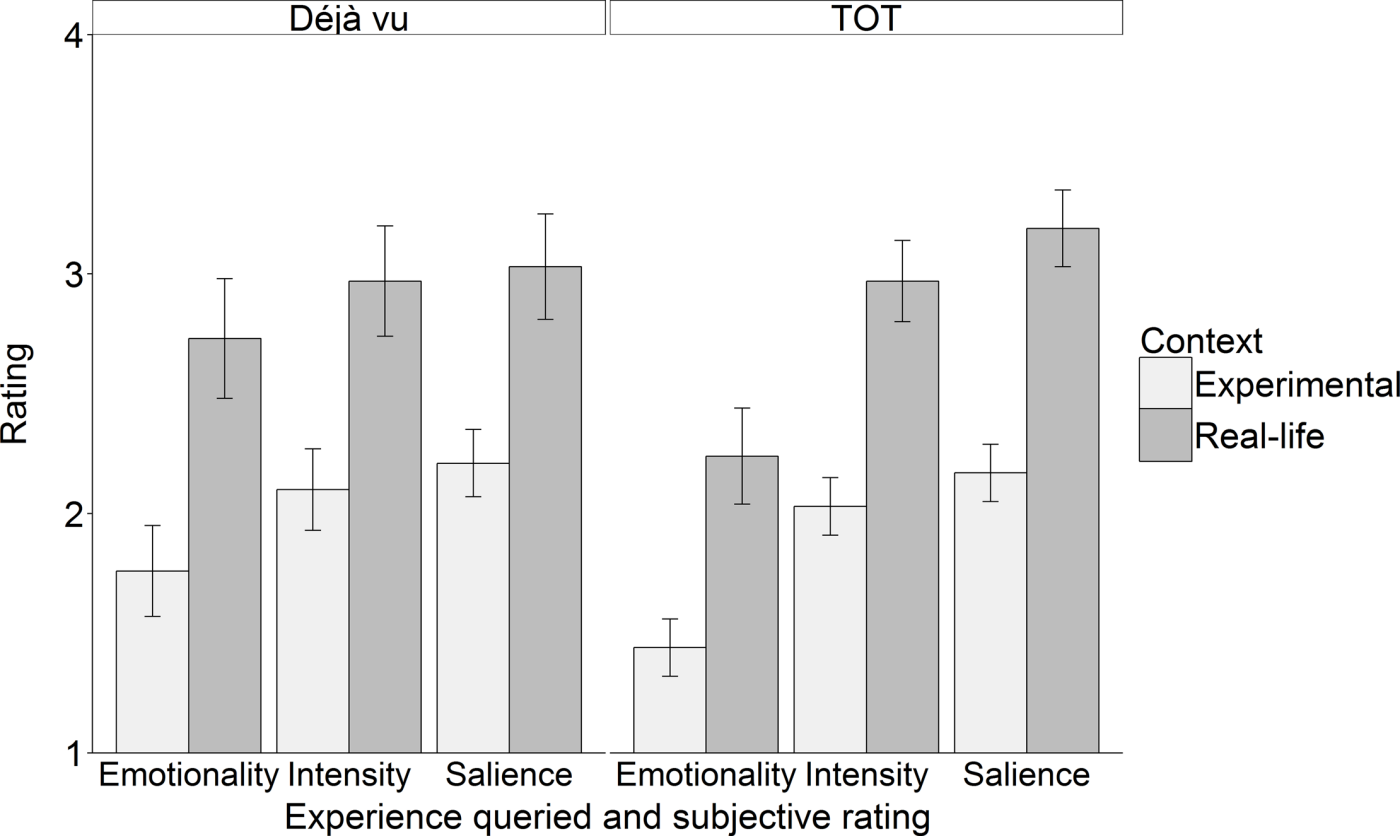
Ratings of experience characteristics across context. Error bars indicate standard errors of the mean.

Lastly, we explored whether participants perceived the experimentally-generated experiences as subjectively different to their real-life experiences. To this end we compared the ratings of salience, intensity and emotionality between reports of real-life and experimentally-generated experiences of déjà vu and TOT in Experiment 2B (see Fig 3). Context (real, experimental) x dimension (salience, intensity, emotionality) repeated measures ANOVAs were used to analyse both déjà vu and TOT reports. For déjà vu reports, there was a main effect of context, meaning real-life déjà vu experiences were rated higher on all dimensions as compared to experimentally-generated déjà vu, *F*(1,28) = 16.17, *p* < .001, *η*_*p*_^*2*^ = .37. There was also a main effect of dimension, *F*(2,56) = 3.56, *p* < .05, *η*_*p*_^*2*^ = .11, suggesting some scales received, overall, higher ratings. TOT reports followed the same pattern with real-life experiences rated higher than experimentally-generated experiences on all dimensions, *F*(1,35) = 55.23, *p* < .001, *η*_*p*_^*2*^ = .61, and with a main effect of dimension, *F*(2,70) = 27.68, *p* < .001, *η*_*p*_^*2*^ = .44, meaning the scales differed on how high participants scored them overall.

We also checked whether the ratings of experimentally-generated experiences differed significantly from 1 (the lowest possible rating) using a one-sample t-test. This was so as to confirm whether these experiences were rated as having any of the three phenomenological characteristics or whether participants simply scored them as phenomenologically neutral. This was confirmed for all ratings (salience, intensity and emotionality) for both déjà vu and TOT (all *p*-values < .001, lowest *t*-value = 3.86).

#### Summary

Asking participants to bring to mind a real-life experience of déjà vu or TOT did not attenuate unexpectedly high reports of their occurrence during the experiment. We replicated a result of Experiment 1 showing an increase in TOT reporting in the third as compared to the first section of the recognition task with no such result observed for déjà vu reporting. Despite the high percentage of both déjà vu and TOT reporters, it is clear that participants perceived experimentally-generated déjà vu and TOT experiences as at least subjectively weaker as compared to their experiences in real-life as revealed by their salience, emotionality and intensity ratings.

These results suggest that the high frequencies of déjà vu and TOT observed in Experiment 1 are likely not due to a lack of clarity about the experiences queried and so might alternatively be linked to the method of questioning employed. More specifically, participants might be led to re-interpret their actual experiences as those they are asked about. We addressed this in Experiment 3 by substituting periodic retrospective questioning for a free report of déjà vu or TOT occurrence, which participants were able to make whenever the experiences occurred.

## Experiment 3

In this experiment we tested whether a different method of questioning would reduce the levels of déjà vu and TOT reported during and after the continuous recognition procedure. In the previous experiments, participants were asked at regular intervals whether they experienced déjà vu or TOT. In this experiment we replaced this recurring question with a button present throughout the entire recognition task, which participants were instructed to press whenever they experienced déjà vu or TOT (depending on their condition). As such we turned from repeatedly assessing retrospectively *whether* a participant had a déjà vu or a TOT experience to a free, real-time report assessment of *when* such an experience occurred. This method of experience assessment would not inevitably reduce responding—it actually provided participants with unlimited opportunity to report the experiences in question (as compared to only being asked 9 times). However, we hypothesised that it should place less pressure on participants toward acquiescence and so lead to a reduction of reports. We thus again repeated the déjà vu and TOT conditions of Experiment 1 with this novel method of querying déjà vu and TOT occurrence. Data from the déjà vu and TOT groups of Experiment 1 once more formed the baseline against which we compared the results of this manipulation.

### Method

#### Participants

A total of 123 participants took part in this experiment, 64 in the Déjà vu condition and 59 in the TOT condition. 41 were women, 76 were men (6 did not disclose their gender) with mean age of 28.9 (*SD* = 8.8; 7 did not disclose age).

#### Materials and procedure

The materials were the same as in Experiment 1. The procedure replicated that of the Déjà vu and TOT conditions in Experiment 1 with one change during the recognition task. Instead of being asked every 12 trials (9 times in total) whether participants experienced déjà vu or TOT, they were presented with a button, onscreen throughout the continuous recognition task, which they could press whenever they had the given experience. At the end of the recognition task, all participants completed the PEQ where they indicated whether at any point during the experiment they experienced déjà vu, TOT or saw any words presented in yellow.

### Results and Discussion

#### Memory performance

To check consistency of performance between groups we again compared sensitivity and bias scores (see Table 1). An independent samples *t*-test revealed that the Déjà vu and TOT groups did not differ on either measure, demonstrating equivalent memory performance on the recognition task in both sensitivity, *t* < 1, and bias, *t*(121) = 1.05, *p* = .30, *d* = .19.

### Post-experimental reports of déjà vu and TOT

**Fig 4:**
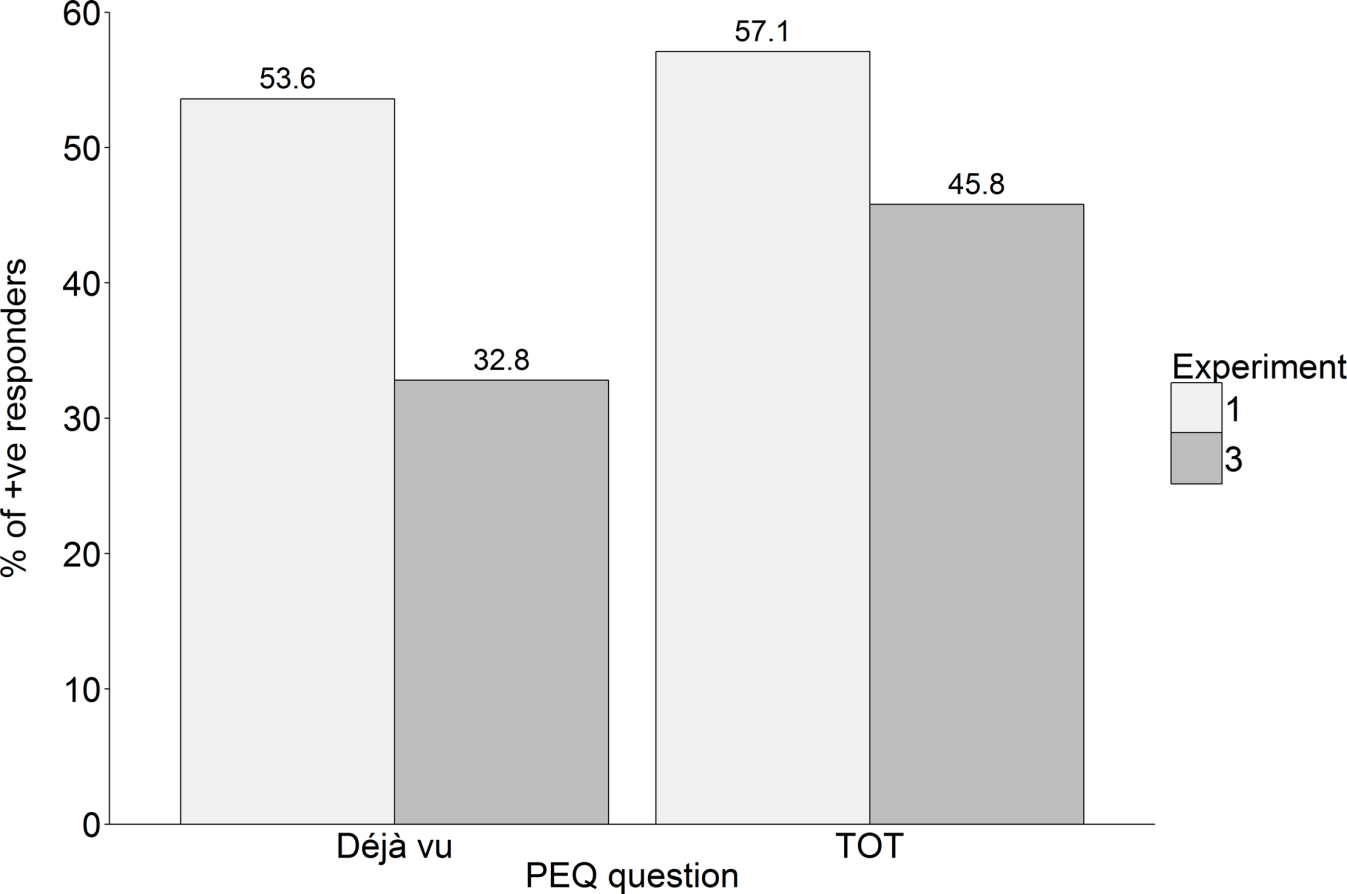
Percentage of participants reporting déjà vu and TOT in the PEQ in Experiments 1 and 3. Déjà vu reports are from Déjà vu conditions and TOT reports are from TOT conditions.

To analyse whether our manipulation had an effect on the percentage of participants reporting déjà vu and TOT experiences we compared déjà vu and TOT reports in this experiment against Déjà vu and TOT reports in the equivalent conditions in Experiment 1 (see Fig 4). There were fewer participants post-experimentally reporting déjà vu in Experiment 3 as compared to Experiment 1, *χ*^*2*^(1) = 5.27, *p* < .05. There was no difference in TOT reports however, *χ*^*2*^(1) = 1.49, *p* = .22.

#### Within-experiment reports of déjà vu and TOT

For those participants who post-experimentally reported experiencing déjà vu or TOT, we examined how many times they reported the given experience during the experiment (see Table 2). More specifically, we analysed whether participants in Experiment 3 reported fewer occurrences of déjà vu and TOT than participants in Experiment 1 during the recognition task. An independent samples *t*-test revealed that participants in the Déjà vu condition of Experiment 1 reported fewer déjà vu experiences during the recognition task than participants in the Déjà vu condition of Experiment 3, *t*(49) = 4.62, *p* < .001, *d* = 1.34. An equivalent analysis of the number of TOT reports also revealed a significant reduction in Experiment 3, *t*(57) = 5.61, *p* < .001, *d* = 1.49.

#### Summary

The free report method of questioning decreased the percentage of participants reporting déjà vu but not TOT relative to Experiment 1. For those participants who post-experimentally reported déjà vu or TOT, we observed a significant decrease in the number of instances of both déjà vu and TOT reported. This is notable as participants here had the opportunity to report *more* experiences than in previous experiments. This shows that using a free report, self-reflective method of questioning, where the participant is asked to continually monitor her subjective state rather than having to retrospectively assess whether an event occurred, may place less pressure on participants toward positive responding. However, the high percentage of participants reporting the sensation post-experimentally still begs the question: what are participants experiencing when they report déjà vu in a standard memory procedure? In Experiment 4, we attempted to capture qualities of the experience most likely to be endorsed by participants. To this end we compared the independent contributions of (i) the term déjà vu and (ii) the operational definition associated with it to subsequent reports of its occurrence.

## Experiment 4

In Experiment 4 we looked at déjà vu reports exclusively. This decision was driven by the flexibility that exists in defining the experience, especially in lay use of the term [15,16]. In the Paramnesia condition, we gave participants the same definition of déjà vu as in previous studies but replaced the label “déjà vu”, which the explanation defined, with “paramnesia”. In the Unpleasant familiarity condition, we kept the label “déjà vu” but only defined the idea of unpleasant familiarity in the explanation (an accepted secondary use of déjà vu in every-day language as confirmed by Merriam-Webster dictionary) [17]. The Paramnesia condition assessed whether it is the definition alone that participants endorse when reporting déjà vu in these experiments. If this is the case, the percentage of reporters should stay the same even without reference to the term “déjà vu”. The Unpleasant familiarity condition assessed whether participants are indicating the experience of mundane familiarity when reporting déjà vu. Across both conditions, the PEQ déjà vu question was kept the same as in previous experiments. We used the same method of questioning as in Experiment 3, favouring free over retrospective reporting. As such we used the déjà vu group of Experiment 3 as the baseline against which we compared results from this experiment. In line with the idea that continuous recognition should generate feelings of familiarity, but should be less likely to generate déjà vu, we predicted that we would see more reports of déjà vu in the Unpleasant familiarity rather than the Paramnesia condition.

### Method

#### Participants

154 participants took part in this study, 78 in the Paramnesia and 76 in the Unpleasant familiarity group. 77 were women and 77 men, mean age 24.6 (*SD* = 7.0; 3 did not disclose age).

#### Materials and procedure

The procedure was a modification of the Déjà vu condition in Experiment 3. In the Paramnesia condition, participants were given the same déjà vu definition as before but this time labelled as *paramnesia*: *Paramnesia is a feeling of familiarity with a situation (e*.*g*. *seeing a word) combined with an awareness that this familiarity is inappropriate (i*.*e*. *you know you have not experienced the situation before)*. The Unpleasant familiarity condition kept the label ‘déjà vu’ but provided an alternative definition: *Déjà vu is the feeling that a situation (e*.*g*. *seeing a word) is overly or unpleasantly familiar*. As in Experiment 3, participants were told to press a button during the recognition task whenever they experienced the subjective state described in the instructions. At the end of the continuous recognition task, they indicated whether they experienced déjà vu, TOT or saw any words presented in yellow at any point in the experiment.

### Results and Discussion

#### Memory performance

As in previous experiments, we first analysed whether participants in the two conditions performed the same on the recognition task (see Table 1). The two groups did not differ in either sensitivity, *t* < 1, or bias, *t*(152) = 1.60, *p* = .11, *d* = .26.

#### Post-experimental reports of déjà vu and TOT

To analyse whether the manipulation had an effect on reporting, we first compared the percentage of post-experimental déjà vu reports across the two groups and the Experiment 3 déjà vu group. The percentage of déjà vu reporters in Experiment 3 (32.8%) did not differ significantly from that reported in the Paramnesia or Unpleasant familiarity conditions (39.7% and 26.3% respectively), *χ*^*2*^(2) = 3.14, *p* = .21.

#### Within-experiment reports of déjà vu and TOT

We also analysed whether the groups differed in how many experiences participants reported on average during the recognition task. For those who reported déjà vu in the PEQ, the number of experiences reported during the recognition task did not differ between the Paramnesia and Unpleasant familiarity conditions and Déjà vu condition of Experiment 3 (see Table 2), *F* < 1.

#### Summary

The lack of difference in déjà vu reports between the Paramnesia condition here and Experiment 3 could suggest that participants were in fact endorsing the déjà vu definition offered to them in their subjective reports, regardless of the label. However, the corresponding absence of a difference across the Unpleasant familiarity condition and Experiment 3 suggests that participants were equally likely to report déjà vu when it was equated with familiarity as when it was equated with the standard definition used in experimental settings. Overall, these results reinforce the idea that participants are less sensitive to what they are being asked as compared to how they are being asked, as manipulated in Experiment 3.

## General Discussion

The subjective nature of déjà vu and TOT means that researchers of these experiences must rely on subjective self-reports. We have no observable identifier or behavioural index of whether the experiences have occurred–an issue as true for behavioural testing as for neurological case studies or surveys of real-life occurrence. As experimenters however, we do have control over how we elicit these self-reports and as such it is crucial to fully appreciate whether our methods influence participant responding (see [2]). Déjà vu and TOT are distinct experiences with unique underlying mechanisms (for reviews see [1,15]) and the methodologies employed for their assessment have therefore developed largely independently of each other. We are not the first to argue that current methods could be improved. It has previously been suggested that déjà vu research should borrow from the procedures used to study TOT, primarily by moving from purely PEQ type reports to trial-level reports [18]. We would argue that a useful parity can be found in TOT research borrowing from practices common in déjà vu research, especially the use of control trials to monitor baseline reporting. All of these concerns are part of a wider, ongoing debate concerning the best practices in the study of all subjective experiences and consciousness. Despite attempts to find objective, no-report measures, self-report remains a crucial tool to assessing inherently first-order, introspective experiences [19]. The purpose of the current study was to establish the extent to which both TOT and déjà vu self-reports are sensitive to the way they are queried with the aim of adding to this debate.

Experiment 1 was designed to assess whether repeated questioning leads to inflated reports of déjà vu and TOT. We expected to find differences in post-experimental responses based on the questions participants were asked during the recognition task. In fact, we found high percentage of déjà vu and TOT reporters no matter the condition. These high levels of déjà vu and TOT reporting are implausible given the absence of any manipulation that could have generated déjà vu or TOT, so in subsequent experiments we attempted to find methods by which déjà vu and TOT reports could be reduced to acceptable levels in the context of no experience having been generated. We used comparisons with real world experiences (Experiments 2A and 2B), changes in the within-experiment questioning method from retrospective to free-report (Experiment 3) and changes in the definition of déjà vu (Experiment 4). Across all experiments, we found unexpectedly high percentage of participants reporting déjà vu and TOT in the PEQ. First we discuss the effects of our manipulations on déjà vu and TOT reports. Next we consider how these elevated false alarms may have come about. Lastly we make recommendations for future research based on the current results.

We found some evidence that the method of questioning used to assess subjective memory experiences is enough to change the percentage of participants reporting its occurrence. In Experiment 1 we observed that, with repeated questioning, more participants reported experiencing TOT in the PEQ than in the other groups. Across experiments that employed the repeated questioning methodology (Experiments 1, 2A and 2B), TOT reports were also more frequent toward the end of the recognition task, after repeated questioning, as compared to the beginning. Further, instructing participants to indicate whenever they experienced déjà vu (Experiment 3) as compared to being repeatedly asked whether it occurred (Experiment 1) reduced the reported number of occurrences of déjà vu and TOT during the experiment and the percentage of participants reporting déjà vu in the PEQ. It is notable that the free report method, which actually allowed participants to report more instances of a given experience, led to fewer reports than the more established retrospective method. In contrast, our manipulations at the instructions stage (Experiments 2A, 2B and 4) had no impact on frequency of reports and percentage of reporters. The results described above suggest research on subjective experiences is more likely to suffer from acquiescence effects rather than lack of agreement or understanding by participants of the experiences under study.

The consistency of reporting across the four experiments has the potential to encourage confidence in déjà vu and TOT self-reports; it could be argued that self-report in the context of research on subjective experiences is a reliable method. Nevertheless, as we stated in the Introduction, the motivation for this paper came from research showing participants report subjective experiences on control trials which were not meant to induce them and which current theory struggles to account for (e.g. [9]). Further, there is clear evidence that participant’s reports are sensitive to task instructions [8] and that participants freely retract experiences reported during study merely when asked to elaborate on their nature [10]. In light of this, the issue of whether consistent self-reports equate to valid self-reports remains.

One interpretation of our findings is that continuous recognition tasks *do* generate déjà vu and TOT. When participants are presented with common nouns on the recognition task, the general familiarity with a word combined with its ‘new’ status on the recognition task could be experienced as déjà vu like. However, this would imply that the simple act of monitoring the *new* vs. *old* status of stimuli is enough to generate the experience. Given the frequent occurrence of such monitoring, both in navigating everyday situations and as a basic requirement in many cognitive tasks, one must conclude that déjà vu should occur very commonly in naturalistic settings and in the multitude of recognition experiments carried out every year. By all accounts this does not seem to be the case. Similarly, one could suggest that seeing a word might have motivated participants to attempt recall of information related to it, which could result in a TOT experience for that word when the retrieval failed. However, in a context where participants are merely asked to indicate whether a word has been seen already or is presented for the first time, the possibility that participants are actively trying to retrieve information about the presented words unrelated to the task at hand and then experience TOTs for this information at the rate that we have observed remains highly unlikely. While we readily acknowledge that *some* participants might have had *some* such experiences, these would not have been generated by our manipulations and we do not believe they could have been produced at the frequency that we observed across the four experiments reported here.

As stated previously, the purpose of using the continuous recognition paradigm was that it was not expected to generate either déjà vu or TOT experiences. The argument made here is not that an elevated report of subjective experiences in experimental settings is problematic. If a manipulation for successful déjà vu or TOT generation has been discovered, then such an elevation is to be expected [18]. We do however believe that we should be wary of paradigms that fail to differentiate between different types of subjective experiences such as familiarity without recollection (as in when somebody seems familiar but we cannot remember where we know them from; see [20]), déjà vu and TOT. Rather than concluding that perhaps this means these experiences are in fact identical, we should ask first why participants might seemingly be conflating them.

We propose that the explanation for the current data is some combination of demand characteristics [5] and the inferential nature of subjective experiences [21]. The current view is that people interpret largely implicit cues (e.g. fluency of processing) in determining whether an item is familiar [22] or whether they are in a subjective mnemonic state such as TOT [21] and that people might experience a degree of uncertainty as to what exactly they are experiencing [23]. Additionally, a large body of literature has demonstrated that the way a question is framed can bias memory reports in eye-witness situations [24], and classic memory [6] and metamemory [25] tasks. If a participant is unsure of what she is experiencing and interprets being asked about experiencing déjà vu or TOT as indicative that she should be experiencing déjà vu or TOT, she may reinterpret her current state as that which she is asked about. This could lead to reports of subjective experiences she is not experiencing. We propose that in making retrospective self-reports, participants are more likely to search for similarities between their actual experience and that which they are asked about. In contrast, in free reports they might initially set a criterion against which they judge whether an event has occurred, thus leaving less room to reinterpret mundane experiences as the experience in question, and therefore produce false alarms.

Another key question of interest is the relationship between real-life and experimentally-generated subjective experiences. Experiment 2B demonstrated that participants perceive whatever they are experiencing in the experiment as different to their experiences in real life. Both déjà vu and TOT were rated as more salient, intense and emotional when experienced in real-life than when experimentally-generated. Similarly, a study that compared naturally occurring and laboratory reported TOTs in the same sample found little to no relationship between the two with the incidence of laboratory TOTs (23 for young adults) far outnumbering the number of naturally occurring TOTs experienced in a 4-week period (5.21 for young adults) [26]. However, one study did find a moderately strong relationship between real-life and experiment-generated experiences in young adults (with no such relationships for older adults) [27] and in another study 90% of participants claimed their laboratory TOTs resemble real-life TOTs [28]. The question of how real-life and experimentally-generated déjà vu compare has only very recently come under scrutiny with data suggesting that naturalistic déjà vu last longer and are more comprehensive (relating to whole scenarios and situations) than experimentally-generated experiences (which tend to be stimulus specific) [10]. More data on the nature of the similarities and differences would be useful.

As to how the presented experiments can inform future empirical work on déjà vu, TOT and related phenomena, the clearest finding appears to be that false alarms may be best reduced by letting participants report whenever the experiences occur, rather than periodically probing them retrospectively. We suggest it is also helpful to include control trials and use participants’ reporting on these as an index of demand characteristics–a common practice in déjà vu research but almost unheard of in TOT studies. Using methods which have been shown to reduce false reporting and using indices which corroborate the effectiveness of such methods will enable greater confidence in the data collected. Asking participants to relate the laboratory induced experiences to their real-life experiences would be a great benefit to the field which frequently discusses this question but to date has done fairly little to collect data to address it. Lastly, building on past research, a post-experimental questionnaire which asks participant to indicate whether, or even how many times, they have experienced déjà vu or TOT during the experiment is good practice and provides an opportunity to corroborate any reports gathered during the experiment. We believe this approach could reduce the presence of false alarms without necessarily promoting extreme conservatism in responding.

Researching experiences such as déjà vu and TOT remains an important endeavour for memory researchers. That the metacognitive assessment of memory retrieval can conflict with or dissociate from the output of that memory system has important implications for our understanding of memory and the higher order cognitive processes monitoring it. However, if participants are confirming the occurrence of the subjective experiences a researcher asks them about simply because they are being asked about them, researchers could inappropriately conclude that i) subjective phenomena are being reported when they are in fact not being experienced; and ii) distinct phenomena such as déjà vu and TOT are manifestations of the same experience. We need methodologies that can reliably tease apart the occurrence of these distinct experiences and that are less susceptible to influence by demand characteristics. Only once these methods have been developed and refined will we reliably be able to study the causes and mechanisms that underlie these curious and compelling mental phenomena.
